# Hormonal and metabolic influences on outcomes in PCOS undergoing assisted reproduction: the role of BMI in fresh embryo transfers

**DOI:** 10.1186/s12884-025-07480-9

**Published:** 2025-03-28

**Authors:** Li Wang, Xiaoyu Yu, Dongsheng Xiong, Mei Leng, Meiyu Liang, Rong Li, Libing He, Heqiu Yan, Xiaoju Zhou, Erniu Jike, Weixin Liu, Jiuzhi Zeng

**Affiliations:** 1 Reproductive Medicine Center, Key Laboratory of Reproductive Medicine, Sichuan Provincial Woman’s and Children’s Hospital, The Affiliated Women’s and Children’s Hospital of Chengdu Medical College, Chengdu, Sichuan 610045 China; 2 School of Laboratory Medicine, Chengdu Medical College, Chengdu, 610000 China

**Keywords:** PCOS, ART, Embryo development, Pregnancy outcomes, Metabolism

## Abstract

**Background:**

This retrospective study aimed to examine the influence of hormonal and metabolic parameters across varying body mass index (BMI) levels on embryo quality and pregnancy outcomes in fresh embryo transfer cycles using assisted reproductive technology (ART) in patients diagnosed with polycystic ovary syndrome (PCOS).

**Methods:**

A total of 167 women diagnosed with PCOS and 266 women without PCOS (control group) were included. Metabolic and hormonal parameters was compared between the two groups to evaluate their relationship with embryo quality and pregnancy outcomes. Subgroup analyses were performed to assess these effects in patients with normal and high BMI.

**Results:**

In the PCOS group, hormonal and metabolic parameters such as insulin, blood lipids, luteinizing hormone (LH), anti-Müllerian hormone (AMH) and antral follicle counting (AFC) were significantly higher than in the control group. The PCOS group also produced more blastocysts and a higher proportion of high-quality blastocysts. However, pregnancy rate and clinical pregnancy rates were similar between the two groups, regardless of BMI. Among the high-BMI PCOS patients, the miscarriage rate was significantly higher compared to the control group, and its rate showed a positive correlation with BMI, LH, and total testosterone (TSTO) levels.

**Conclusion:**

Hormonal imbalances and glucose-lipid metabolism have minimal influence on embryo development in PCOS patients. However, hormonal factors-particularly in PCOS patients with high BMI—significantly influence pregnancy outcomes, with elevated BMI and androgen levels increasing the risk of miscarriage. These findings underscore the importance of addressing metabolic and hormonal factors in the management of PCOS patients undergoing ART.

## Introduction

Polycystic ovary syndrome (PCOS) is a multifaceted endocrine and metabolic disorder affecting women of reproductive age [1]. Clinically, it is characterized by hyperandrogenemia, hormonal imbalances, ovulatory dysfunction, and polycystic ovarian morphology [2]. Metabolic abnormalities, including obesity, dyslipidemia, and insulin resistance, are commonly observed in PCOS patients, presenting significant challenges for both clinicians and patients [3]. The manifestations of hormonal and metabolic disturbances in PCOS patients vary considerably across different body mass index (BMI) levels. Obese PCOS patients are more likely to exhibit significant metabolic dysfunctions, whereas those with a normal BMI tend to present with pronounced hormonal imbalances [4, 5]. BMI serve as a critical variable that influences both metabolic and hormonal profiles, as well as its role in ART [6]. 

The pathogenesis of PCOS is complex, involving an interplay of environmental, genetic, and endocrine factors. While ART has become a primary therapeutic approach for PCOS, its success relies on a comprehensive understanding of the metabolic and hormonal factors that influence embryo quality and pregnancy outcomes [7]. Dysregulation of glycolipid metabolism and hormonal imbalances are key considerations in ART for PCOS patients, as hyperandrogenism and insulin resistance may negatively affect embryonic development and pregnancy outcomes [8, 9]. In recent years, improvements in ART outcomes have underscored the need for further investigation into these underlying mechanisms.

ART is traditionally considered the definitive treatment for PCOS and is typically pursued only after women with PCOS fail to conceive through ovulation induction [10]. The heterogeneous of PCOS, including various in obesity, hyperinsulinemia, and low-grade chronic inflammation, significantly impacts reproductive outcomes [11]. However, existing research has primarily focused on pregnancy complications such as gestational diabetes and hypertension, while the impact of BMI, glycolipid metabolism, and hormone levels on embryonic development and pregnancy outcomes in PCOS patients remains insufficiently explored [12, 13]. This study aims to address these gaps by analyzing the metabolic characteristics of PCOS patients with normal and high BMI. Specifically, we hypothesize that BMI, along with hormonal and metabolic parameters, significantly influences embryonic development and pregnancy outcomes in fresh embryo transfer cycles. By identifying key influencing factors, we seek to provide theoretical insights and practical references for clinical applications.

## Materials and methods

### Study design

This study included 167 women diagnosed with PCOS at the Reproductive Medicine Center and 266 healthy women of reproductive age undergoing in vitro fertilization and embryo transfer (IVF-ET) due to fallopian tube disorders or male infertility. Participants were recruited from Sichuan Women and Children’s Hospital between January 2021 and September 2023. The inclusion criteria for the PCOS group consisted of women aged 20–37 who met the Rotterdam diagnostic criteria for PCOS, which requires the presence of at least two of the following conditions: oligomenorrhea or amenorrhea, elevated androgen levels or clinical manifestations of hyperandrogenism, and sonographic evidence of polycystic ovarian morphology. Additionally, all participants had indications for IVF/ICSI and underwent superovulation induction using a gonadotropin-releasing hormone antagonist (GnRH-ant) regimen. The control group included women aged 20–37 with infertility due to fallopian tube issues or male factor infertility, normal ovarian reserve, or regular ovulatory menstrual cycles. They also underwent superovulation induction and IVF/ICSI. The exclusion criteria included a diagnosis of PCOS, endometriosis, untreated or uncorrected endocrine disorders or significant systemic diseases. To minimize potential confounding factors such as age and metabolic comorbidities, participants were matched by age and BMI as closely as possible. Standardized protocols were followed for ovarian stimulation, embryo grading, and pregnancy outcomes assessment to ensure consistency in data collection and analysis.

### Ethical approval

All procedures and protocols were approved by the Ethics Committee of Sichuan Provincial Woman and Children’s Hospital (Approval No. 20231012-245). Informed consent was obtained from all participants, and the study was conducted in accordance with the guidelines of the Declaration of Helsinki.

### Laboratory tests

Venous blood samples were collected using vacuum pipettes following a 12-hour fasting period. Serum samples were subsequently transferred into standard gel separation tubes for biochemical analysis and processed within one hour of collection. Levels of fasting plasma glucose (FPG), triglycerides (TG), total cholesterol (TC), low-density lipoprotein cholesterol (LDL), and high-density lipoprotein cholesterol (HDL) levels were measured using a Hitachi 008AS automatic analyzer (Hitachi, Tokyo, Japan). Hormonal assays were performed to assess estradiol (E2), testosterone (TSTO), prolactin (PRL), progesterone (P), and follicle-stimulating hormone (FSH) levels.

### Ovarian stimulation protocols

Both PCOS patients and controls underwent controlled ovarian stimulation (COS) using a GnRH-ant regimen as part of their reproductive treatment. On the second day of the menstrual cycle, an individualized dosing strategy was implemented to optimize follicular development, enhance success rates, and minimize complications. Endocrine hormone levels and ultrasound assessments were performed, and the initial gonadotropin dose was determined based on BMI, hormone levels, and ovarian reserve markers (AMH and AFC). Due to their increased ovarian sensitivity, PCOS patients generally received a lower starting dose, whereas control group participants were administered doses based on established clinical guidelines [14]. 

Follicular development was monitored via transvaginal ultrasound from stimulation days Gn4-5, alongside serum E2, LH and P measurements. Gonadotropin dosages were adjusted based on ovary responses. When follicles reached a diameter of 12–14 mm or serum E2 level exceeded 400pg/ml, a GnRH-antagonist was administered subcutaneously until the day of human chorionic gonadotropin (HCG) injection. Final oocyte maturation was triggered when serum E2 levels plateaued, with at least 1–3 follicles exceeding 18 mm in diameter and at least half of the follicles reaching 14 mm or greater. Oocyte maturation is triggered using either hCG alone (8,000–10,000 IU) or a combination of hCG (2,000 IU) and a GnRH agonist (GnRHa, 0.2 mg).

### Oocytes retrieval and fertilization

Following the trigger, all follicles exceeding 10 mm were aspirated via transvaginal ultrasound-guided puncture. Approximately six hours after oocyte retrieval, either conventional IVF or ICSI was performed based on semen parameters. Fertilization was assessed 16–18 h post-insemination, with successful fertilization defined by the presence of two pronuclei, two polar bodies in the perivitelline space, and two nuclei within the cytoplasm. On day 3, embryos were cultured to the cleavage stage and evaluated for quality.

### Embryo grading and quality evaluation

Cleavage-stage embryos were assessed on day 3 based on cell morphology, uniformity, cytoplasmic granularity, and fragmentation rate. High-quality cleavage-stage embryos were defined as those containing 6–8 blastomeres and classified as graded I or II. Blastocysts were evaluated using the Gardner grading system, with assesses the inner cell mass and trophoblast layer.

Patients who met the transplantation criteria with the same cycle underwent fresh embryo transfer. Progesterone supplementation was administered post-transfer and continued until the 10th week of gestation.

### Outcomes measures

Clinical pregnancy was defined as the presence of a gestational sac at 7 weeks post-embryo transfer (ET). Miscarriage was classified as pregnancy loss occurring 12 weeks of gestation (early miscarriage) or between 12 and 24 weeks (late miscarriage). Preterm birth was defined as delivery between 28 and 37 weeks of gestation. Live birth was defined as the delivery at least one live infant.

### Statistical analysis

Statistical analyses were performed using SPSS 22.0 (IBM, Chicago, USA). Continuous variables were presented as mean ± standard deviation (SD) for normally distributed data and as median (interquartile range [IQR]) for non-normally distributed data. Group comparisons were performed using an independent-sample t-test for normally distributed data and the Mann-Whitney U test for non-normally distributed data, ensuring appropriate handling of parametric and non-parametric distributions in embryo quality and pregnancy outcome analyses. Correlation analyses were conducted using Pearson or Spearman correlation coefficients, depending on the normality of the data, to evaluate associations between PCOS, embryo quality, and pregnancy outcomes. A significance level of *P* < 0.05 was considered statistically significant.

## Results

### Study population

The study included a total of 433 participants, comprising 167 patients diagnosed with PCOS and 266 control subjects, ages between 20 and 37 years. There was no statistically significant difference in age between the two groups (*P* = 0.179). However, the PCOS group had a significantly longer duration of infertility (*P* < 0.001) and exhibited higher BMI (*P* < 0.001) compared to the control group (Table 1).

**Table 1:** The baseline data of the 433 assessed women

Variables	PCOS (*n* = 167)	Control (*n* = 266)	*P*-value
Age (years)	29.22 ± 3.34	29.66 ± 3.32	0.179
Duration of infertility (years)	3.00 (2.00, 5.00)	2.05 (1.90, 4.00)	< 0.001
BMI(kg/m^2^)	23.96 ± 3.50	22.61 ± 3.51	< 0.001
FPG (mmol/L)	5.10 ± 0.54	5.10 ± 0.42	0.954
Insulin (µIU/ml)	11.55 (8.53, 15.38)	9.19 (6.18, 13.28)	< 0.001
HOMA-IR	2.55 (1.89, 3.69)	2.08 (1.40, 3.06)	< 0.001
HDL (mmol/L)	1.36 (1.15, 1.55)	1.40 (1.25, 1.63)	0.008
LDL (mmol/L)	2.77 ± 0.75	2.40 ± 0.68	< 0.001
TC (mmol/L)	4.68 ± 0.87	4.38 ± 0.81	< 0.001
TG (mmol/L)	1.28 (0.86, 1.90)	0.91 (0.68, 1.30)	< 0.001
E_2_ (pg/ml)	35.08 (25.20, 47.30)	34.16 (26.54, 42.40)	0.195
LH (mIU/ml)	7.09 (4.88, 10.81)	4.42 (3.23, 5.72)	< 0.001
FSH (mIU/ml)	6.19 (5.50, 7.30)	6.33 (5.43, 7.50)	0.368
P (ng/ml)	0.40 (0.26, 0.54)	0.44 (0.30, 0.58)	0.087
PRL (µIU/ml)	306.90 (225.58, 420.60)	305.26 (232.70, 403.86)	0.150
TSTO (ng/ml)	0.37 (0.25, 0.49)	0.23 (0.15, 0.33)	< 0.001
AMH (ng/ml)	5.71 (3.81, 8.56)	2.42 (1.64, 3.61)	< 0.001

FPG, fasting blood glucose; HOMA-IR, homeostatic model assessment of insulin resistance; TG, triglyceride; TC, total cholesterol; LDL, low-density lipoprotein; HDL, high-density lipoprotein; E_2_, Estradiol; TSTO, testosterone; PRL, prolactin; FSH, follicular stimulating hormone; LH, luteinizing hormone; P, progesterone; AMH, anti-Müllerian Hormone

Metabolic analysis indicated no significant difference in fasting plasma glucose (FPG) levels between the two groups (*P* = 0.954). However, insulin levels and the homeostatic model assessment of insulin resistance (HOMA-IR) were significantly higher in the PCOS group. Additionally, the PCOS group exhibited elevated levels of low-density lipoprotein (LDL, *P* < 0.001), total cholesterol (TC, *P* < 0.001), and triglycerides (TG, *P* < 0.001), while high-density lipoprotein (HDL) levels were lower (*P* = 0.008) compared to controls (shown in Table 1).

Hormonal analysis revealed that the levels of luteinizing hormone (LH, *P* < 0.001), testosterone (TSTO, *P* < 0.001), and anti-Müllerian hormone (AMH, *P* < 0.001) were significantly higher in the PCOS group. In contrast, no significant differences were observed between the two groups for follicle-stimulating hormone (FSH, *P* = 0.368), estradiol (E2, *P* = 0.195), progesterone (*P* = 0.087), and prolactin (PRL, *P* = 0.150) levels (Table 1).

### Comparison of COS conditions and pregnancy outcomes

A comparative analysis of follicular and embryo quality between the PCOS and control groups revealed significant differences (Table 2). The antral follicle count (AFC) was notably higher in the PCOS group (*P* < 0.001), whereas no significant differences were found in the distribution of primary and secondary infertility diagnoses. The proportion of ICSI procedures was significant greater in the control group (*P* = 0.011). Regarding ovulation induction parameters, the control group had a slightly longer stimulation duration (*P* = 0.016) and required significantly higher initial and total gonadotropin (Gn) dose (*P* < 0.001). Serum E2 levels on the day of HCG administration were elevated in the PCOS group (*P* < 0.001), though the number of retrieved oocytes did not differ significantly (*P* = 0.097). However, the PCOS group exhibited a significantly higher number of metaphase II (MII) follicles (*P* = 0.026), while the number of fertilized two-pronuclear (2PN) zygotes remained comparable between the groups (*P* = 0.312) (Table 2).

**Table 2:** Comparison of COS conditions and pregnancy outcomes between PCOS and control group

Variables	PCOS (*n* = 167)	Control (*n* = 266)	*P*-value
Antral follicle count (AFC)	24.00 (20.00, 29.00)	14.00 (10.00, 18.00)	< 0.001
Infertility diagnosis			
Primary infertility (%)	62.87 (105/167)	59.40 (158/266)	0.471
Secondary infertility, (%)	37.13 (62/167)	40.60 (108/266)	0.471
ICSI cycles (%)	15.57 (26/167)	25.94 (69/266)	0.011
Duration of stimulation (days)	10.42 ± 2.12	10.91 ± 2.00	0.016
Gn priming dosage (IU)	150.00 (125.00, 175.00)	187.50 (150.00, 225.00)	< 0.001
Total dose of Gn (IU)	1650.00 (1350.00, 2100.00)	2250 (1650.00, 2775.00)	< 0.001
E2 on hCG day (pg/ml)	2546.29 (1809.50, 3460.50)	1957.00 (1353.82, 2701.72)	< 0.001
Retrieved oocytes	11.00 (9.00, 15.00)	11.00 (8.00, 14.00)	0.097
No. of MII oocytes	9.00 (7.00, 13.00)	8.00 (6.00, 11.00)	0.026
Fertilized oocytes (2PN)	6.00 (4.00, 8.00)	6.00 (4.00, 8.00)	0.312
No. of available embryos of Day 3	6.00 (4.00, 9.00)	5.00 (4.00, 7.00)	0.005
No. of blastocysts formed	2.00 (0.00, 5.00)	2.00 (0.00, 4.00)	0.247
Rate of blastocyst formed (%)	75.00 (50.00, 87.00)	70.00 (50.00, 100.0)	0.388
No. of high quality blastocysts	0.00 (0.00, 1.00)	0.00 (0.00, 0.00)	0.003
Rate of high quality blastocysts (%)	25.00 (0.00, 50.00)	0.00 (0.00,33.00)	0.023
No. of embryos transferred	2.00 (2.00, 2.00)	2.00 (2.00, 2.00)	0.840
Endometrial thickness (mm)	10.58 ± 2.35	11.70 ± 2.69	< 0.001
Pregnancy rate, (%)	62.87 (105/167)	62.41(166/266)	0.922
Clinical pregnancy rate (%)	47.90 (80/167)	52.26 (139/266)	0.378
Miscarriage rate (%)	23.75 (19/80)	10.79 (15/139)	0.011
Live birth rate (%)	36.53 (61/167)	46.62 (124/266)	0.039

Notably, embryo development outcomes indicated that the PCOS group had a higher number of available D3 embryos (*P* = 0.005) and a significantly greater number and proportion of high-quality blastocysts (*P* = 0.003 and *P* = 0.023, respectively), although the total number of blastocysts formed was similar between groups (*P* = 0.247). The endometrial thickness on the day of embryo transfer was significantly lower in the PCOS group (*P* < 0.001). Despite these differences, pregnancy rates (*P* = 0.922) and clinical pregnancy rates (*P* = 0.378) were comparable. An unexpected finding was the significantly higher miscarriage rate in the PCOS group (*P* = 0.011), which contributed to a notably lower live birth rate (*P* = 0.039) (shown in Table 2). These results suggest that although the increased proportion of high-quality blastocysts in PCOS patients indicates that their oocyte developmental potential remains preserved under controlled ovarian stimulation, this did not translate into improved pregnancy outcomes. The higher miscarriage rate and lower live birth rate suggest that factors beyond embryo quality—such as endometrial receptivity, metabolic disturbances, and hormonal imbalances—may play a critical role in pregnancy maintenance in this population [5]. 

### Correlation analysis of hormonal and metabolic indicators with PCOS and pregnancy outcomes

PCOS is a multifaceted endocrine disorder that impacts both hormonal and metabolic health, potentially influencing reproductive outcomes. To better understand these relationships, a correlation analysis was conducted. We found that PCOS was positively correlated with LH (*r* = 0.453, *P* < 0.001), TSTO (*r* = 0.350, *P* < 0.001), and AMH (*r* = 0.588, *P* < 0.001). Additionally, PCOS exhibited positive correlations with insulin (*r* = 0.204, *P* < 0.001), HOMA-IR (*r* = 0.190, *P* < 0.001), TC (*r* = 0.174, *P* < 0.001), TG (*r* = 0.263, *P* < 0.001), and LDL (*r* = 0.252, *P* < 0.001). In contrast, PCOS was negatively correlated with FSH, HDL (*r* = -0.128, *P* < 0.008). Furthermore, significant correlations were observed between PCOS and several embryonic parameters, including the number of available Day 3 (D3) embryos (*r* = 0.134, *P* = 0.005), good quality embryos of Day 3 (*r* = 0.172, *P* < 0.001), good quality blastocysts (*r* = 0.145, *P* = 0.002), and high-quality rate of blastocyst (*r* = 0.150, *P* = 0.023). Furthermore, miscarriage rates were positively correlated with PCOS (*r* = 0.183, *P* = 0.007), BMI (*r* = 0.157, *P* = 0.021) and LH levels (*r* = 0.160, *P* = 0.018). These findings suggest that both hormonal and metabolic alterations in PCOS are associated with embryonic development and pregnancy outcomes, including miscarriage rates.

### Comparison of hormone and metabolic indicators in high-BMI individuals

Obesity is a common condition among patients with PCOS, further complicating their metabolic and reproductive health. Among obese individuals (BMI ≥ 24), the PCOS group had a significantly longer duration of infertility compared to the control group (*P* = 0.011), despite no significant difference in BMI between the groups (*P* = 0.155). Metabolic analysis revealed that LDL levels were significantly higher in the PCOS group (*P* = 0.035). However, no significant differences were observed in FPG, insulin, HDL, TC and TG (Table 3). This suggests that PCOS is associated with an increased risk of dyslipidemia, even among high-BMI women, potentially influenced by individual factors. Hormonal analysis showed that the PCOS group exhibited significantly higher level of LH, TSTO, AMH and E2 compared to the control group (Table 3). However, no significant differences were found in FSH, P, and PRL. The elevated AMH levels reinforce its role as a biomarker of ovarian dysfunction in PCOS, indicating an increased follicular reserve but also potential ovulation dysfunction. Additionally, the higher LH levels further highlight the disrupted reproductive endocrine profile in PCOS (Table 3).

**Table 3:** The baseline data of the high-BMI assessed women

Variables	PCOS (*n* = 77)	Control (*n* = 100)	*P*-value
Age (years)	29.38 ± 3.64	30.24 ± 3.48	0.110
Duration of infertility (years)	4.00 (2.00, 5.00)	3.00 (1.00, 4.00)	0.011
BMI(kg/m^2^)	26.92 ± 2.66	26.41 ± 2.11	0.155
FPG (mmol/L)	5.17 ± 0.61	5.21 ± 0.47	0.309
Insulin (µIU/ml)	13.81 (10.81, 21.29)	13.37 (9.77, 18.06)	0.203
HOMA-IR	3.17 (2.36, 5.18)	3.05 (2.33, 4.27)	0.302
HDL (mmol/L)	1.27 (1.04, 1.43)	1.30 (1.13, 1.49)	0.068
LDL (mmol/L)	2.91 ± 0.82	2.68 ± 0.73	0.035
TC (mmol/L)	4.68 ± 0.94	4.49 ± 0.97	0.145
TG (mmol/L)	1.53 (0.98, 2.18)	1.22 (0.89, 1.72)	0.072
E_2_ (pg/ml)	36.96 (25.70, 47.36)	29.85 (22.29, 39.89)	0.009
LH (mIU/ml)	6.77 (4.78, 10.75)	3.60 (2.63, 5.22)	< 0.001
FSH (mIU/ml)	6.00 (5.28, 6.91)	6.10 (5.14, 7.25)	0.724
P (ng/ml)	0.41 (0.23, 0.51)	0.39 (0.27, 0.55)	0.823
PRL (µIU/ml)	299.70 (212.51, 374.50)	284.65 (231.65, 392.17)	0.842
TSTO (ng/ml)	0.38 (0.23, 0.48)	0.23 (0.15, 0.34)	< 0.001
AMH (ng/ml)	6.29 (3.70, 8.13)	2.39 (1.65, 3.52)	< 0.001

FPG, fasting blood glucose; HOMA-IR, homeostatic model assessment of insulin resistance; TG, triglyceride; TC, total cholesterol; LDL, low-density lipoprotein; HDL, high-density lipoprotein; E_2_, Estradiol; TSTO, testosterone; PRL, prolactin; FSH, follicular stimulating hormone; LH, luteinizing hormone; P, progesterone; AMH, anti-Müllerian Hormone

Despite the well-established association between PCOS and insulin resistance, no significant differences in FPG and insulin levels were observed between the two groups. This finding highlights the heterogeneity in metabolic profiles among high-BMI PCOS patients, emphasizing the need for personalized clinical assessment in this population.

### Comparison of COS conditions and pregnancy outcome in high-BMI individuals

Obesity is widely recognized as a significant factor embryo quality [15]. Table 4 compares COS conditions and pregnancy outcomes between high-BMI individual with and without PCOS. The PCOS group had a significantly higher AFC (*P* < 0.001), required lower Gn priming (*P* = 0.001), and received a reduced total dose of Gn (*P* = 0.001). The retrieved oocyte count (*P* = 0.043) and MII oocyte numbers (*P* = 0.022) were also higher. However, no significant differences were observed in E2 and P levels on HCG day.

**Table 4:** Comparison of COS conditions and pregnancy outcomes in high-BMI individuals

Variables	PCOS (*n* = 77)	Control (*n* = 100)	*P*-value
Antral follicle count (AFC)	24.00 (20.00, 29.50)	14.50 (10.00, 19.00)	< 0.001
Infertility diagnosis			
Primary infertility (%)	61.04 (47/77)	55.00 (55/100)	0.420
Secondary infertility (%)	38.96 (30/77)	45.00 (45/100)	0.420
ICSI cycles (%)	16.88 (13/77)	24.00(24/100)	0.248
Duration of stimulation (days)	10.57 ± 2.41	11.28 ± 2.38	0.052
Gn priming dosage (IU)	150 (150, 200.00)	.. 200.00 (150.00, 225.00)	0.001
Total dose of Gn (IU)	1800 (1500, 2400)	2256.25 (1800.00, 3168.90)	0.001
E2 on hCG day (pg/ml)	2566.50 (1818.50, 3164.25)	1813.93 (1173.50, 2408.75)	< 0.001
Retrieved oocytes	11.00 (9.00, 15.00)	10.00 (7.00, 13.75)	0.043
No. of MII oocytes	10.00 (8.00, 13.00)	8.00 (6.00, 11.00)	0.022
Fertilized oocytes (2PN)	6.00 (4.50, 9.00)	6.00 (4.00, 7.75)	0.292
No. of available embryos of Day 3	6.00 (4.00, 9.00)	5.00 (4.00, 7.00)	0.025
No. of blastocysts formed	2.00 (0.00, 5.00)	1.00 (0.00, 4.00)	0.242
Rate of blastocyst formed (%)	75.00 (50.00, 90.00)	71.00 (33.00, 90.00)	0.347
No. of high quality blastocysts	0.00 (0.00, 1.00)	0.00 (0.00, 0.00)	0.056
Rate of high quality blastocysts (%)	22.00 (0.00, 48.00)	0.00 (0.00, 37.00)	0.326
No. of embryos transferred	1.83 ± 0.41	1.89 ± 0.31	0.263
Endometrial thickness (mm)	10.78 ± 2.54	11.76 ± 2.75	0.017
Pregnancy rate (%)	61.04 (47/77)	68.00 (68/100)	0.336
Clinical pregnancy rate (%)	49.35 (38/77)	53.00 (53/100)	0.630
Miscarriage rate (%)	31.58 (12/38)	13.20 (7/53)	0.033
Live birth rate (%)	33.77 (26/77)	46.00 (46/100)	0.100

Regarding embryonic development, no significant differences were found in fertilized oocytes (*P* = 0.292), blastocyst formation rate (*P* = 0.347), or high-quality blastocyst rate (*P* = 0.326). However, the PCOS group had more available embryos on day 3 (*P* = 0.025), suggesting better embryo potential. Although the number of high-quality blastocysts was slightly higher, the differences was not statistically significant (*P* = 0.056). This implies that high-BMI PCOS patients may still produce viable embryos under controlled ovarian stimulation. While pregnancy rate (*P* = 0.336), clinical pregnancy rate (*P* = 0.630), and live birth rate (*P* = 0.100) did not differ significantly between the groups, the miscarriage rate was notable higher in the PCOS group (*P* = 0.033), indicating potential risks related to implantation or endometrial receptivity (shown in Table 4).

### Correlation analysis of hormonal and metabolic indicators with high-BMI PCOS and pregnancy outcomes

Obesity has been suggested to influence both embryo quality and pregnancy outcomes, potentially through its effects on hormonal and metabolic processes. To further investigate these relationships, we also conducted a correlation analysis among women with a BMI ≥ 24.0. The results revealed that PCOS was positively correlated with E2 (*r* = 0.197, *P* = 0.009), LH (*r* = 0.479, *P* < 0.001), TSTO (*r* = 0.355, *P* < 0.001) and AMH (*r* = 0.552, *P* < 0.001). Additionally, PCOS was positively correlated with the number of retrieved oocytes (*r* = 0.153, *P* = 0.043), fertilized oocytes (*r* = 0.154, *P* = 0.041), the number of Day 3 (D3) available embryos (*r* = 0.168, *P* = 0.025), and good quality embryos of Day 3 (*r* = 0.149, *P* = 0.048). Among individuals with obesity, miscarriage rates were positively correlated with BMI (*r* = 0.231, *P* = 0.028), LH (*r* = 0.244, *P* = 0.020) and TSTO (*r* = 0.249, *P* = 0.017) levels.

### Comparison of hormone and metabolic indicators in normal-BMI individuals

In individuals with a BMI < 24, the PCOS group exhibited a significantly higher BMI (*P* < 0.05) and greater metabolic disturbances compared to the control group, including elevated insulin, HOMA-IR, LDL, TC, and TG levels (*P* < 0.01). However, no significant differences were observed in FPG and HDL levels between the two groups (*P* > 0.05). Hormonal analysis revealed significantly higher levels of LH, TSTO, and AMH in the PCOS group (*P* < 0.01), while FSH, P, PRL and E2 levels were comparable between groups (*P* > 0.05) (shown in Table 5). Despite the presence of hyperinsulinemia in the PCOS group, FPG and HDL levels did not differ significantly, suggesting potential compensatory mechanisms in glucose regulation (Table 5). These findings highlight the complex metabolic and hormonal profile in normal-BMI individuals with PCOS, as well as long-term risks metabolic disturbances and ovarian dysfunction.

**Table 5:** The baseline data of the normal-BMI assessed women

Variables	PCOS (*n* = 90)	Control (*n* = 166)	*P*-value
Age (years)	29.08 ± 3.08	29.31 ± 3.17	0.577
Duration of infertility (years)	2.50 (1.37, 5.00)	2.00 (2.00, 4.00)	0.285
BMI(kg/m^2^)	21.43 ± 1.65	20.33 ± 1.79	< 0.001
FPG (mmol/L)	5.04 ± 0.47	5.04 ± 0.38	0.981
Insulin (µIU/ml)	10.48 (7.87, 12.38)	7.67 (5.52, 10.58)	< 0.001
HOMA-IR	2.30 (1.73, 2.83)	1.64 (1.20, 2.37)	< 0.001
HDL (mmol/L)	1.47 (1.24, 1.66)	1.50 (1.31, 1.69)	0.218
LDL (mmol/L)	2.64 ± 0.66	2.18 (1.85, 2.58)	< 0.001
TC (mmol/L)	4.69 ± 0.80	4.31 ± 0.69	< 0.001
TG (mmol/L)	1.16 (0.80, 1.61)	0.81 (0.64, 1.07)	< 0.001
E_2_ (pg/ml)	33.92 (24.10, 46.42)	36.41 (28.98, 44.80)	0.285
LH (mIU/ml)	7.11 (4.91, 11.21)	4.54 (3.69, 5.85)	< 0.001
FSH (mIU/ml)	6.56 (5.63, 7.60)	6.44 (5.63, 7.72)	0.727
P (ng/ml)	0.39 (0.28, 0.60)	0.46 (0.33, 0.59)	0.061
PRL (µIU/ml)	314.50 (257.02, 443.92)	315.30 (232.77, 416.85)	0.358
TSTO (ng/ml)	0.34 (0.25, 0.50)	0.24 (0.15, 0.33)	< 0.001
AMH (ng/ml)	6.31 (3.84, 9.09)	2.46 (1.59, 3.72)	< 0.001

FPG, fasting blood glucose; HOMA-IR, homeostatic model assessment of insulin resistance; TG, triglyceride; TC, total cholesterol; LDL, low-density lipoprotein; HDL, high-density lipoprotein; E_2_, Estradiol; TSTO, testosterone; PRL, prolactin; FSH, follicular stimulating hormone; LH, luteinizing hormone; P, progesterone; AMH, anti-Müllerian Hormone

### Comparison of COS conditions and pregnancy outcomes in normal-BMI individuals

In individuals with a BMI < 24, the PCOS group required significantly lower initial and total doses of gonadotropins and had a shorter duration of ovulation induction compared to the control group (*P* < 0.001, Table 6). Consistent with findings in high-BMI individuals, the normal-BMI PCOS group showed better outcomes in terms of the number of fertilized oocytes, available embryos on day 3 (D3), and blastocyst formation. Specifically, the normal-BMI PCOS group exhibited a higher number and rate of high-quality blastocysts, with statistically significant improvement (*P* < 0.05, Table 6). However, no significant differences were observed between the PCOS and control groups in clinical pregnancy outcomes, including pregnancy rates, clinical pregnancy rates, miscarriage rates, or birth rates. These findings suggest that, despite the improved embryo quality, the normal-BMI PCOS may not experience corresponding advantages in clinical pregnancy outcomes.

**Table 6:** Comparison of COS conditions and pregnancy outcomes in normal-BMI individuals

Variables	PCOS (*n* = 90)	Control (*n* = 166)	*P*-value
Antral follicle count (AFC)	24.00 (19.00, 29.25)	14.00 (10.00, 17.25)	< 0.001
Infertility diagnosis			
Primary infertility (%)	64.44 (58/90)	62.05 (103/166)	0.705
Secondary infertility (%)	33.56 (32/90)	37.95 (63/166)	0.705
ICSI cycles (%)	14.44 (13/90)	27.11 (45/166)	0.021
Gn duration (days)	10.00 (9.00, 11.50)	11.00 (9.00, 12.00)	0.041
Gonadotropin priming dosage (IU)	150.00 (125.00, 150.00)	168.75 (150.00, 225.00)	< 0.001
Gonadotropin dosage (IU)	1500.00 (1350.00, 1800.00)	2100.00 (1525.00, 2556.25)	< 0.001
E2 on hCG day (pg/ml)	2546.29 (1744.25, 3653.75)	2064.50 (1475.25, 2851.00)	0.001
Retrieved oocytes	10.00 (8.75, 14.00)	11.00 (8.00, 14.00)	0.671
No. of MII oocytes	9.00 (6.00, 12.25)	8.50 (6.00, 11.00)	0.384
Fertilized oocytes (2PN)	6.00 (4.00, 8.00)	6.00 (4.00, 8.00)	0.653
No. of available embryos of Day 3	6.00 (4.00, 9.00)	5.50 (4.00, 7.25)	0.077
No. of blastocysts formed	2.00 (0.00, 5.25)	2.00 (0.00, 4.00)	0.507
Rate of blastocyst formed (%)	77.00(50.00, 87.00)	70.00 (50.00, 100.00)	0.618
No. of high quality blastocysts	0.00 (0.00, 1.00)	0.00 (0.00, 1.00)	0.017
Rate of high quality blastocysts (%)	31.00 (0.00, 50.00)	0.00 (0.00, 33.00)	0.032
No. of embryos transferred	1.91 ± 0.38	1.85 ± 0.39	0.227
Endometrial thickness (mm)	10.31 ± 2.00	11.66 ± 2.66	< 0.001
Pregnancy rate (%)	64.44 (58/90)	59.04 (98/166)	0.397
Clinical pregnancy rate (%)	46.67(42/90)	51.81 (86/166)	0.432
Miscarriage rate (%)	16.67(7/42)	9.30 (8/86)	0.224
Live birth rate (%)	38.89 (35/90)	46.99 (78/166)	0.213

### Correlation analysis of hormonal and metabolic indicators with normal-BMI PCOS and pregnancy outcomes

We further examined the associations between metabolic and hormonal factors on reproductive outcomes in individuals with a BMI < 24. Our analysis revealed a positive correlation between PCOS and BMI (*r* = 0.289, *P* < 0.001). Additionally, significant positive associations were observed between PCOS and hormonal factors, including LH (*r* = 0.446, *P* < 0.001), TSTO (*r* = 0.348, *P* < 0.001), and AMH (*r* = 0.615, *P* < 0.001). PCOS also showed positive correlations with metabolic indicators such as insulin (*r* = 0.271, *P* < 0.001), TC (*r* = 0.211, *P* < 0.001), TG (*r* = 0.334, *P* < 0.001), and LDL (*r* = 0.285, *P* < 0.001). Furthermore, PCOS was positively correlated with key embryo quality indicators, including the number of high-quality embryos on D3 (*r* = 0.186, *P* = 0.003), the number of high-quality blastocysts (*r* = 0.150, *P* = 0.017), and the high-quality blastocyst rate (*r* = 0.181, *P* = 0.031). However, in individuals with a BMI < 24, no significant correlation was found between miscarriage rates and metabolic, hormonal, or embryo quality factors.

## Discussion

Obesity is a key factor affecting glycolipid metabolism in PCOS patients [16]. Adiposity leads to substantial alterations in serum hormone levels and metabolic parameters compared to non-obese PCOS patients [17]. Our analysis demonstrates that HIGH-BMI individuals with PCOS exhibit more pronounced hormonal dysregulation, characterized by elevated levels of insulin, LH, TSTO, and AMH. In contrast, changes in lipid metabolism are primarily limited to increased LDL levels in the PCOS group. Among normal-BMI individuals, PCOS is associated with disruptions in both glucose and lipid metabolism, alongside hormonal disturbances, as evidenced by elevated insulin, LDL, TC, TG, LH, TSTO, and AMH. In previous studies, we identified TG as an independent risk factor for PCOS, correlating with lipid metabolism [4]. Given the increasing prevalence of infertility (8-15%), ART has become a crucial intervention, particularly for PCOS patients indergoing prolonged treatment [18]. While ovulation induction is typically based on ovarian reserve, obese PCOS patients exhibits substantial variability in their response to stimulation and embryo quality. Advances in ovarian stimulation protocols have identified BMI, hormone levels, and ovarian reserves as key parameters for predicting outcomes [19, 20]. Cycles segmentation is now considered as the gold standard for IVF in PCOS patients. However, fresh cycles remain widely used, necessitating further investigation into their effects on embryo development and pregnancy success [21]. This study primarily focuses on the indicators of fresh cycles in eligible PCOS patients to provide insights in embryo developments and pregnancy outcomes.

Research on follicle quality, fertilization success, embryo availability, and pregnancy outcomes in PCOS across different BMI levels remains limited. In this study, we compared ovulation induction, embryo quality, and pregnancy outcomes between PCOS and individuals without the condition. Consistent with previous research, our findings show that PCOS patients required lower gonadotropin doses due to their elevated AFC and AMH levels, which influence ovarian response and embryo development [22, 23]. PCOS patients exhibited fertilization similar to non-PCOS patients, with no significant differences in oocytes retrieval, fertilization rate, or formation of two-pronuclear (2PN) zygotes. However, On Day 3 (D3), the PCOS group had more available embryos, a higher number of blastocysts, and an increased blastocyst formation rate, suggesting a strong ovarian response and an expanded ovarian reserve. These findings indicated that existing evidence does not support the use of oocyte support the use of [24]. Correlation analysis further revealed a positive association between PCOS and the number of available blastocysts on D3, as well as the rate of high-quality blastocysts, reinforcing the idea that PCOS does not necessarily compromise embryo development. While these results highlight the reproductive potential of PCOS patients, the relationship between oocyte and embryo quality remains debated, with some studies suggesting potential deficits in oocyte competence despite an increased number of retrieved oocytes. These conflicting perspectives emphasize the need for further research to clarify the impact of PCOS on embryo viability and IVF success rates across diverse patient profiles [25, 26]. 

Given the complex pathogenesis of PCOS, pregnancy outcome indicators—such as clinical pregnancy rate, miscarriage rate, and live birth rate—are crucial for assessing treatment efficacy [27]. Despite a significant higher number of oocytes retrieved from PCOS patients during IVF or ICSI, oocyte quality may remain suboptimal. However, most studies report no significant difference in assisted pregnancy outcomes between PCOS and non-PCOS patients undergoing ICSI/IVF [28, 29]. Our findings align with this, demonstrating that while PCOS patients exhibit a robust ovarian reserve, this does not necessarily translate into higher success rate in cycle transplantation [28, 30]. Previous research suggests that women with PCOS may alter the phenotype and functionality of the endometrium through increased endocrine and metabolic activities, thereby elevating the risk of miscarriage and preterm delivery, and contributing to adverse pregnancy outcomes [31]. Both the intrauterine maternal environment and endometrial receptivity are vital for embryonic development. Metabolic and hormonal abnormalities, including obesity, dysregulated glucose metabolism, and insulin resistance, disrupt the internal environment, which can negatively affect embryo quality [32, 33]. 

BMI significantly influences reproductive outcomes in PCOS by exacerbating metabolic and hormonal imbalances that impair folliculogenesis, oocyte quality, endometrial receptivity, and increase the risk of implantation failure [27, 34]. Considering the impact of obesity on PCOS, our observations show that both obese and non-obese PCOS patients exhibit higher numbers of fertilized oocytes, Day 3 embryos and blastocysts. With the insulin and lipid profiles (TC, TG, and LDL) showed stronger correlations with PCOS, while miscarriage rates were higher in PCOS patients, highlighting the role of adiposity in adverse pregnancy outcomes. Obesity contributes to increased miscarriage rates in PCOS by exacerbating chronic inflammation, oxidative stress, and hormonal imbalances. Elevated levels of pro-inflammatory cytokines impair endometrial receptivity and placental development, while excessive insulin and androgens disrupt the delicate endocrine. Furthermore, metabolic dysregulation affects uterine blood flow and immune tolerance, further increasing the of risk miscarriage [35–37]. 

Given the impact of high BMI on ART outcomes in PCOS patients, early intervention in weight management and lifestyle modifications is critical. A 5–10% reduction in BMI can significantly improve ovulation, endometrial receptivity, and implantation [38]. Addressing insulin resistance, restoring hormonal and metabolic balance, and optimizing BMI can enhance ART success rates. These findings underscore the importance of integrating metabolic analysis and BMI into clinical guidelines for personalized fertility treatment. Given the elevated risk of miscarriage in obese PCOS patients, targeted interventions should be included in ART programs to improve pregnancy outcomes and reduce complications [39]. In clinical practice, early metabolic screening and weight management should also be prioritized to improve fertility and overall health in PCOS patients [40]. 

## Conclusion

Our study confirms a correlation between metabolic and hormonal abnormalities in PCOS patients and their reproductive outcomes, with embryo quality appearing superior in PCOS cases. However, significant disparities in clinical pregnancy outcomes were observed between obese and non-obese patients. Obesity is a key modifiable factor associated with increased miscarriage risk, highlighting the need for targeted interventions. Clinically, incorporating metabolic management into fertility treatment could improve pregnancy outcomes in PCOS patients. Early lifestyle interventions and weight management programs are crucial for mitigating reproductive risks associated with PCOS. However, this study is limited by its sample size and retrospective design, warranting further large-scale prospective studies to validate these findings and refine clinical guidelines.

## Data Availability

The data that support the findings of this study are available from the corresponding author upon reasonable request (zengjiuzhi9@163.com).
